# Exposure of *Culicoides sonorensis* to Enzootic Strains of Bluetongue Virus Demonstrates Temperature- and Virus-Specific Effects on Virogenesis

**DOI:** 10.3390/v13061016

**Published:** 2021-05-28

**Authors:** Jennifer Kopanke, Justin Lee, Mark Stenglein, Molly Carpenter, Lee W. Cohnstaedt, William C. Wilson, Christie Mayo

**Affiliations:** 1 Department of Microbiology, Immunology, and Pathology, Colorado State University, Fort Collins, CO 80523, USA; jennifer.kopanke@wsu.edu (J.K.); psd8@cdc.gov (J.L.); mark.stenglein@colostate.edu (M.S.); molly.carpenter@colostate.edu (M.C.); 2 Arthropod-Borne Animal Diseases Research Unit, United States Department of Agriculture—Agricultural Research Service, Manhattan, KS 66502, USA; lee.cohnstaedt@usda.gov; 3 National Bio and Agro-Defense Facility (NBAF), United States Department of Agriculture—Agricultural Research Service, 1880 Kimball Ave, Suite 300 CGAHR, Manhattan, KS 66502, USA; William.wilson2@usda.gov

**Keywords:** bluetongue virus, *Culicoides sonorensis*, vector, arbovirus, reassortment, temperature, virogenesis

## Abstract

Bluetongue virus (BTV) is a segmented RNA virus transmitted by *Culicoides* midges. Climatic factors, animal movement, vector species, and viral mutation and reassortment may all play a role in the occurrence of BTV outbreaks among susceptible ruminants. We used two enzootic strains of BTV (BTV-2 and BTV-10) to explore the potential for *Culicoides sonorensis*, a key North American vector, to be infected with these viruses, and identify the impact of temperature variations on virogenesis during infection. While BTV-10 replicated readily in *C. sonorensis* following an infectious blood meal, BTV-2 was less likely to result in productive infection at biologically relevant exposure levels. Moreover, when *C. sonorensis* were co-exposed to both viruses, we did not detect reassortment between the two viruses, despite previous in vitro findings indicating that BTV-2 and BTV-10 are able to reassort successfully. These results highlight that numerous factors, including vector species and exposure dose, may impact the in vivo replication of varying BTV strains, and underscore the complexities of BTV ecology in North America.

## 1. Introduction

Arboviruses represent one of several important types of pathogens anticipated to increase in range and frequency with the progression of climate change [1,2]. These viruses are transmitted by arthropod vectors and represent an emergent disease threat to both human and animal populations. Bluetongue virus (BTV), the type species of genus *Orbivirus* (family *Reoviridae*), is an arbovirus transmitted by biting midges of the *Culicoides* genus (Diptera: Ceratopogonidae). BTV can affect both wild and domestic ruminants and severe disease is characterized by clinical signs reflective of the virus’s ability to cause profound vasculitis [3]. Animals may develop high fevers, edema, coronitis, mucosal erosions, and respiratory distress, etc. [4]. Production declines, animal losses, and trade restrictions contribute to the significant economic impact of BTV [5].

Bluetongue virus has classically been distributed throughout much of the tropics and subtropics, with seasonal circulation in more temperate regions ranging from approximately 35° South to 40° North [6,7]. The virus’s range is defined by the presence of one or more of a large number of competent vector species of the genus *Culicoides*. More than 1300 species of *Culicoides* exist worldwide, but to date only approximately 30 species have been demonstrated to transmit BTV [8,9,10]. Of the numerous species of *Culicoides* present in North America (>150), only a handful are considered to be key BTV vectors [11,12]. These include *Culicoides sonorensis*, which is the predominant BTV vector in North America, and *Culicoides insignis* [13,14,15,16]. While *C. sonorensis* is distributed throughout much of the US, *C. insignis* localizes to the southeast US, predominantly Florida [17].

BTV serotype is defined by the VP2 outer capsid protein, which is the major antigenic determinant of this virus. Of the currently described serotypes of BTV, *C. sonorensis* and *C. insignis* are implicated in the spread of five enzootic serotypes found in North America: BTV-2, BTV-10, BTV-11, BTV-13, and BTV-17 [18]. BTV-3 is currently considered a serotype exotic to the US, but recently has established a presence throughout the southeastern and central regions of the country [19].

The VP2 protein is encoded by one of the 10 genomic segments of double-stranded RNA (segment 2) that make up the BTV genetic structure. The segmented genome of BTV provides this virus and related viruses an additional mechanism of genetic diversification beyond the accumulation of mutations or homologous recombination. Reassortment is a phenomenon that can occur during coinfection, where progeny viruses inherit genome segments from more than one parent virus [20]. Reassortment plays an important role in the overall genetic diversification of BTV and its relatives, as has been demonstrated both in vitro and in vivo [21,22,23,24,25,26,27,28]. While reassortment is known to occur in both the insect vector and ruminant hosts, the features that contribute to and modulate the occurrence of this phenomenon are only somewhat understood.

Various groups have shown that climatic conditions such as temperature have an important effect on vector life history parameters and the rate of BTV virogenesis in the vector, with higher temperatures being associated with more rapid BTV replication [29,30,31,32]. This is important for a number of reasons in terms of ensuring accurate predictive strategies and mitigation efforts (i.e., predicting BTV circulation during peak temperature seasons), in addition to assessing the potential impacts of progressive climate change. Warmer climates are likely to result in shifts in vector distributions and vector competence, which may enhance BTV transmission [3,6,33].

The impacts of higher temperatures promoting faster rates of virogenesis is well characterized in *C. sonorensis* midges and previous studies have documented that reassortment in *C. sonorensis* is reportedly higher (42%) than in vertebrate hosts (5% in sheep) [21,29,33]. However, the impact of temperature on viral reassortment in the vector is unknown. More rapid virogenesis at higher temperatures could lead to higher rates of co-infection of individual cells and consequently increased rates of viral reassortment. The potential for extensive BTV reassortment and its association with temperature could significantly impact surveillance and mitigation strategies. Moreover, such an interaction would affect how BTV may spread to naive populations and the likelihood of reassortant BTV viruses causing disease in animals in otherwise enzootic areas. To test our hypothesis that higher temperatures would drive increased rates of BTV virogenesis, and potentially reassortment, in the vector, we exposed laboratory-reared *C. sonorensis* to two enzootic BTV strains (BTV-2 and BTV-10), either as single virus exposures or co-exposures, and subsequently reared midges at three different temperatures.

## 2. Materials and Methods

### 2.1. Viruses

BTV-10 California 1952 (strain 8, ATCC VR-187) and BTV-2 Florida 1982 (ATCC VR-983) were obtained from ATCC and had been passed 8 and 4 times in BHK 21 cells, respectively [34,35,36]. Whole genome sequences of each virus were previously determined by our lab (GenBank accessions—BTV-2: MW456737–MW456746; BTV-10: MW456747–MW456756) [37].

Infectious titers were estimated via a 50% tissue culture infectious dose (TCID50). Briefly, 10-fold dilutions of each virus were prepared and 50 µL of each dilution was introduced in triplicate to a 96-well microtitration plate. BHK 21 cells were added (1.55 × 10^4^ cells per well) along with 50 µL EMEM, and the virus and cells were incubated at 37 °C with 5% CO_2_ for 96 h. At 96 h, cells were stained with a crystal violet solution, and an infectious titer of each virus was determined using the Reed-Muench equation [38].

### 2.2. BTV-2 and BTV-10 Infection in Culicoides Cell Line

The CuVaW3 line is derived from *C. sonorensis* embryos from the Ausman colony, which was isolated in Weld Co., Colorado [39]. Cells were maintained as previously described with Schneider’s insect medium supplemented with 15% fetal bovine serum, 0.0006% (*w*/*v*) reduced glutathione, 0.003% (*w*/*v*) L-asparagine, 0.0018% of 10 mg/mL bovine insulin, 1% of 200 mM L-glutamine, 0.21% (*w*/*v*) sodium hydroxide, 0.06% (*w*/*v*) calcium chloride, 5% cell culture grade water, 0.4% HCl (12.1N), and 0.04% sodium bicarbonate to maintain the pH at 6.7 [40]. One-step viral growth curves were performed for each virus at a multiplicity of infection (MOI) of ~0.2 TCID50. BTV-2 or BTV-10 was used to inoculate confluent monolayers of CuVaW3 cells in duplicate. One mL of inoculum was added to each flask (25 cm^2^) and incubated for 1 h at 27 °C with frequent rocking. An additional mL of maintenance media was added to each flask after incubation, and infected cells were maintained at 27 °C with no CO_2_ supplementation. Five hundred microliters of viral supernatant were collected from each flask at 2, 6, 12, 24, 48, 72, and 96 h post-inoculation and immediately stored at −80 °C until TCID50 assays could be performed.

### 2.3. Culicoides Maintenance and Infection

*C. sonorensis* from the AK colony (isolated in Idaho in 1973 and maintained at USDA ARS, Manhattan, KS, USA) were obtained from USDA ARS and allowed to acclimate for at least 24 h at 25 °C on a 12:12 light cycle with 10% sugar water provided *ad libitum* prior to being exposed to BTV via a virus-spiked blood meal [41,42]. *C. sonorensis* were 3–4 days post-emergence at feeding.

Defibrinated sheep blood (Hemostat Laboratories, Dixon, CA, USA or Lampire Biological Laboratories, Everett, PA, USA) was confirmed to be negative for BTV virus and antibodies via qRT-PCR and cELISA (VMRD, Pullman, WA, USA), respectively. Blood was then spiked with BTV and was made available to *Culicoides* in glass bell feeders through parafilm membranes. During feeding, blood was maintained at 37 °C. *Culicoides* were allowed to feed for 90 min to maximize the number of blood-fed females. Following this, *Culicoides* were chilled at −20 °C for 5 min and then sorted into groups using a modified chill table. Only blood-fed females were retained. These were divided into groups of several hundred *Culicoides* per container based on BTV exposure (BTV-2 only, BTV-10 only, BTV-2+10, and negative; Table 1). Five to ten blood-fed females were immediately harvested from each group and screened for uptake of virus via BTV qRT-PCR to confirm that *Culicoides* had successfully been exposed to the respective viruses.

Containers were made of non-treated paper tubes (Rigid Paper Tube Corporation, Wayne, NJ, USA) with sheer pantyhose over the lid to permit air exchange and feeding. Sugar water (10% *w*/*v*) was available at all times via a cotton wick in each container. *Culicoides* were offered a BTV-negative blood meal for ~30 min every 3–4 days as above and were maintained at one of three temperatures (20, 25 or 30 °C) with a 12:12 light cycle for the remainder of the experiment.

Initial experiments were performed in two parts due to limitations in the number of *Culicoides* that could be obtained and housed at one time. BTV-2+10 co-exposure experiments were performed first (Experiment 1), followed by BTV-2 and BTV-10 single-virus exposures and survival experiments (Experiment 2) (Table 1). The same BTV stocks were used for each iteration of experiments, and negative control groups were included with each.

Survival experiments were performed in duplicate for each virus (BTV-2, BTV-10, BTV-2+10, negative) at each temperature. Groups of 50 *Culicoides* per container were maintained at the same temperatures (20, 25 or 30 °C) as experimental groups, but were only used to count the number of surviving midges each day. No midges were collected for plaque assays or qRT-PCR from the survival groups.

### 2.4. Culicoides Collections

Following initial BTV exposure, subsets of blood-fed females were collected over the course of 2 to 3 weeks. Our goal was to track BTV virogenesis via qRT-PCR across temperatures and time, in addition to determining whether the temperature at which midges were held (20, 25 or 30 °C) would affect the generation of reassortant BTV.

For both singly exposed (BTV-2 or BTV-10) and co-exposed (BTV-2+10) groups, 5 *Culicoides* were collected in triplicate from each temperature every other day for qRT-PCR analysis until there were no midges remaining. Additionally, starting on day 3 and then continuing every 4 days until the end of the experiment, groups of 10 midges from the BTV-2+10 co-exposed group were collected in triplicate from each temperature for plaque assays. The qRT-PCR analysis was also performed on these midge pools. Five midges from the control group were collected approximately weekly to ensure that they remained BTV-negative throughout the course of the study. After collection, *Culicoides* were immediately processed for plaque assays (BTV-2+10 pools only) or were stored at −80 °C until the qRT-PCR analysis.

In both cases, midges were vigorously homogenized with a sterile pestle in Eagle’s Minimum Essential Medium (EMEM) at a volume of 50 µL per midge (i.e., 250 µL for groups of 5 midges and 500 µL for groups of 10 midges). Homogenates were centrifuged briefly, and then 50 µL of supernatant was collected for qRT-PCR and stored at −80 °C until RNA extractions were performed. For the co-exposed groups of 10 midges, 400 µL of homogenate was sterile-filtered (0.22 µM Millex-GV syringe filter, MilliporeSigma, Burlington, MA, USA) and diluted further in EMEM at 1:2, 1:10, 1:100, 1:1000, and 1:10,000 dilutions for plaque assays, which were performed immediately after collection.

### 2.5. Plaque Assays

BHK 21 cells were seeded in 6-well plates 48 h prior to setting up plaque assays (1.0 × 10^5^ cells/well). Cells were maintained in EMEM with 10% heat-inactivated fetal bovine serum (FBS), 10% tryptose phosphate broth, and 1% penicillin streptomycin (10,000 U/mL). Cells were kept at 37 °C with 5% CO_2_ supplementation.

Confluent monolayers were washed once with PBS pH 7.4 prior to inoculation with dilutions of *Culicoides* homogenate. Five hundred microliters of each dilution were added to a well and incubated for 1 h at 37 °C with frequent rocking to disperse the virus. After incubation, the inoculum was removed and cells were washed once with PBS pH 7.4, followed by overlay with 2 mL of 3:1 BHK media:2% agarose in Earle’s Buffered Salt Solution (EBSS). Plates were incubated at 37 °C for 96 h or until the plaques became evident. At this time, 1 mL of overlay (3:1 BHK media:2% agarose in EBSS with ~0.1% neutral red stain) was added. Plaques were picked 8–24 h after the second overlay when plaques were visibly apparent. Agarose plugs were taken from well-isolated plaques and were propagated in individual wells of a 24- or 48-well plate with BHK 21 cells (4.65 × 10^4^ per well). The supernatant was harvested when cytopathic effects (CPE) were advanced. The harvested viruses were promptly stored at −80 °C until further analysis.

### 2.6. Nucleic Acid Extraction and DNase Treatment

Nucleic acids were extracted from *Culicoides* homogenates and viral supernatants using Applied Biosystem’s MagMAX RNA/DNA Pathogen kit (Invitrogen, Carlsbad, CA, USA) according to the manufacturer’s instructions. Extractions were performed either manually or using the KingFisher Flex robot (Thermo Fisher, Waltham, MA, USA).

Extracted insect homogenates were treated with DNase 1, RNase free (Thermo Fisher). Briefly, 12 µL of extracted nucleic acid from each sample was treated with 2 µL of DNase 1 and 2 µL of 10X buffer. Samples were incubated at 37 °C × 30 min, and then 2 µL of EDTA was added to each sample and heated at 65 °C × 10 min to inactivate the DNase.

### 2.7. qRT-PCR

RNA was subsequently screened in duplicate for the presence of BTV using a universal BTV qRT-PCR that detects BTV segment 10 as previously described using SuperScript III One-step qRT-PCR reagents (Thermo Fisher) at half-reaction volumes [43,44].

To normalize any variations in extraction efficiency, the qRT-PCR based on *Culicoides* mitochondrial cytochrome *c* oxidase subunit 1 (*cox*1) was also performed in duplicate for each sample. Primers were selected based on previously published work (BFculicFm and C1-N-2191) and a HEX-based probe (3′HEX-TGAATACTT/ZEN/CCTCCTTCTCTTTCTT -3IABkFQ/5′, Integrated DNA Technologies, Coralville, IA, USA) was designed based on GenBank sequences of this gene using Geneious v.10.2.2 [45,46,47]. SuperScript III One-step qRT-PCR reagents and volumes were the same as those used for BTV qRT-PCR, except the samples did not undergo the initial 95 °C denaturation step in the presence of primers, as was performed for BTV [44]. Appropriate positive and negative controls for both BTV and *Culicoides cox*1 were run with each plate. A no-reverse transcriptase (no-RT) control was run to confirm that the DNAse treatment was effective (i.e., no amplification of *cox*1 in the absence of reverse transcriptase).

To ensure that the BTV qRT-PCR and *Culicoides cox*1 qRT-PCR were comparable, we ran side-by-side qRT-PCRs in triplicate on serial dilutions of extracted, DNase treated, BTV-infected midges to determine the relative efficiencies of each primer/probe set. Both targets had similar slopes and efficiencies (R^2^ = 0.99 for both BTV and *cox*1) under the qRT-PCR conditions used (Supplementary Figure S1).

BTV Ct values from pools of midges were normalized by *Culicoides cox*1 using the ΔCt method based on mean Ct values for BTV and *cox*1 for each sample (Ct_BTV_ − Ct*_cox_*_1_ = ΔCt_normalized_). Values were expressed as −ΔCt in certain figures to improve the interpretation of data.

In these midge pools, productive BTV virogenesis was considered to exist when *cox*1-normalized BTV Ct values dropped below the baseline BTV ΔCt level (determined as the mean normalized BTV Ct value from insects collected immediately post-blood meal (day 0): ΔCt = 7.5). Linear regressions were used to analyze the rate of virogenesis for each virus and temperature (GraphPad Prism v. 8.0). Samples with undetectable BTV via qRT-PCR were excluded from these calculations. Regressions were calculated based on −ΔCt values starting the day where BTV copy numbers were at their lowest point to account for variations in detectable RNA.

### 2.8. BTV Segment-Specific Sequencing of Plaques

The genotype of individual plaques propagated from co-exposed insects was then determined using a novel, amplicon-based sequencing approach that can rapidly distinguish between BTV-2 and BTV-10 across all 10 segments of genomic dsRNA. As described elsewhere, we used a two-step PCR approach to create amplicons of regions of each BTV-2 and BTV-10 segment that could be differentiated by several non-homologous nucleotides within each amplified sequence [37]. Briefly, 2 µL of nucleic acid from propagated viruses (isolated from BTV-2+10 co-exposed *Culicoides*) was used as input. Primer combinations and concentrations were as those previously described, except with slight variations in round one primer concentrations (Supplementary Table S1) [37]. Bioinformatics analysis and processing were also performed as described in Kopanke et al. [37]_._ The reads mapping to each parental strain were quantified and used to determine the presence of reassortment in progeny viruses (presence of reads mapping to one or more segments from both parental strains). Due to the presence of low-level mis-mapping of reads between BTV-2 and BTV-10 for certain segments in the amplicon assay, only plaques with >90% of all reads mapping to one parent segment or the other were included in our final analyses. Plaques with multiple missing segments and/or very low sequence coverage (i.e., those that did not get reads across all 10 segments) were also excluded from downstream analyses. In some cases, only segment 2 did not receive sufficient sequencing reads. In these cases, a BTV serotype-specific qRT-PCR was performed on the extracted plaques [48].

### 2.9. Exposure of Culicoides to High Titer BTV-2

Since we observed that midges exposed to lower doses of BTV-2 did not seem to replicate the virus, we attempted to determine whether *C. sonorensis* could be successfully infected with higher titers of BTV-2. Using the same design as Experiments 1 and 2 and with the same stocks of BTV-2, we exposed smaller groups of *C. sonorensis* to the virus at titers 30- and 50-fold higher than our initial dose (BTV-2 LO: 3.06 × 10^6^ and BTV-2 HI: 5.1 × 10^6^ TCID50/mL, respectively). BTV-exposed midges (n = 127 for BTV-2 LO; n = 87 for BTV-2 HI) were then reared as described above. Duplicate groups of 50 midges exposed to the 30-fold (low) and 50-fold (high) dose were also maintained to monitor survival at the different titers. Experiments were terminated at day 14, as our earlier experiments had shown that midges held at 25 °C demonstrated productive virogenesis several days before this time point.

At days 7, 11, and 14 post-blood meal, we harvested pools of 5 midges from each group (BTV-2 LO and BTV-2 HI) for screening via BTV qRT-PCR. At day 14, a group of 5 midges from each group was homogenized in EMEM and filtered as described above for plaque assay preparation. Insect homogenates were subsequently used to inoculate confluent 25 cm^2^ flasks of BHK 21 cells. Briefly, the *Culicoides* homogenate was diluted in EMEM to reach a total volume of 1 mL, which was used to inoculate cell monolayers. The monolayers were incubated along with the virus for 1 h at 37 °C, and then an additional 4 mL of maintenance media was added to each flask. Flasks were incubated for 96 h and monitored for the development of CPE daily. If flasks developed CPE, the virus and cells were harvested for extraction and BTV qRT-PCR as described above. The BTV serotype was confirmed using serotype-specific, segment 2-based primers and probe [48].

As midges in this experiment were exposed to much higher titers of BTV than those in our initial experiments, the day 0 BTV ΔCt values for these pools were markedly lower. Therefore, for these midge pools, productive BTV virogenesis was considered to occur when *cox*1-normalized BTV Ct values approached the day 0 baseline BTV ΔCt level.

### 2.10. Statistics

Linear regressions and statistical analyses including one-way ANOVA and two-way ANOVA were performed using GraphPad Prism v. 8.0. Additionally, we performed log-rank Mantel-Cox and did multiple comparisons with a Bonferroni adjustment on survival analysis data. Values where *p* < 0.05 were considered significant.

## 3. Results

### 3.1. BTV-2 and BTV-10 In Vitro Replication Kinetics

Previous results from our lab show that BTV-2 and BTV-10 grow similarly and are able to reassort in BHK 21 cells [37]. However, since this may be a cell-type specific phenomenon, we opted to examine the replication kinetics of these viruses in CuVaW3 cells as a way to better estimate the overall susceptibility of *C. sonorensis* to BTV-2 and BTV-10 [39].

**Figure 1:**
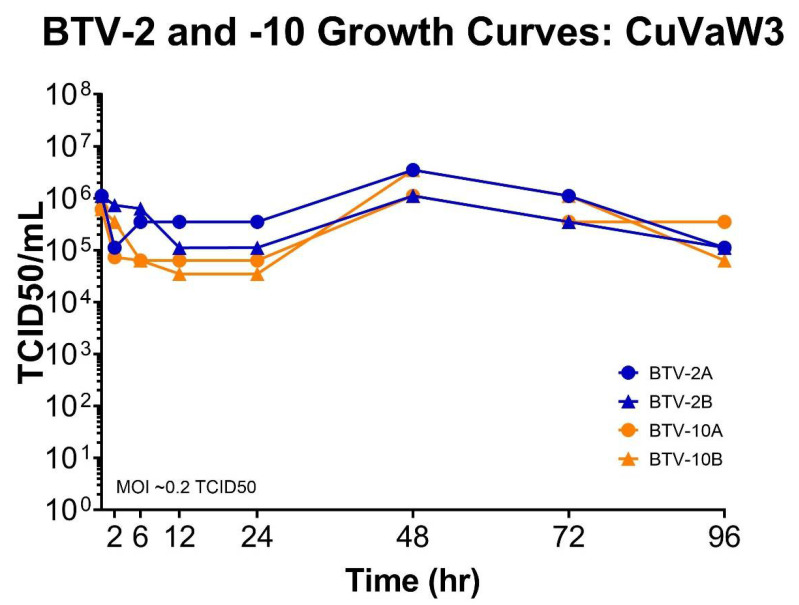
BTV-2 and BTV-10 growth kinetics on CuVaW3 cells. BTV-2 and BTV-10 were used to initiate infections on CuVaW3 cells in duplicate. BTV-2 replicates (BTV-2A or BTV-2B) are shown by blue lines connecting circles or triangles; BTV-10 replicates (BTV-10A or BTV-10B) are shown by orange lines connecting circles or triangles. Cell monolayers were inoculated with an MOI of 0.2 TCID50. Supernatant was collected at 2, 6, 12, 24, 48, 72, and 96 h post infection. TCID50s were performed at each time point to determine the titer.

When infected at an MOI of ~0.2 TCID50, CuVaW3 cells supported similar growth kinetics for both BTV-2 and BTV-10 (Figure 1).

### 3.2. Temperature Effect on BTV Replication in C. sonorensis

**Figure 2:**
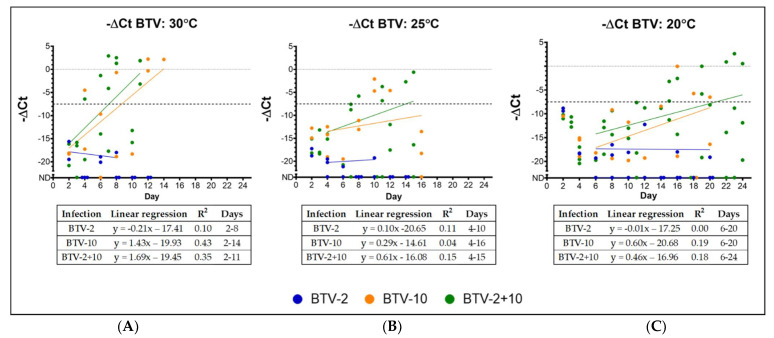
BTV virogenesis in midges held at 30, 25, and 20 °C. Each point indicates a single pool of *Culicoides*, and ΔCt values are presented. ΔCt is calculated as the difference between mean BTV Ct values and *cox*1 Ct values for each sample. To make graphs more intuitive, −ΔCt values are presented. BTV-2 samples are represented in blue; BTV-10 samples are represented in orange; BTV-2+10 samples are represented in green. The dashed line indicates mean post-blood meal day 0 ΔCt across all infection groups (BTV-2, BTV-10, and BTV-2+10). Points depicted at “ND” (not detected) indicate undetectable BTV Ct values and were not included in linear regressions. (**A**) BTV virogenesis in midges held at 30 °C: BTV virogenesis is evident in pools of *C. sonorensis* exposed to BTV-10 and BTV-2+10 at 4 days after blood meal when held at 30 °C. BTV-2 remains near undetectable limits across all days. (**B**) BTV virogenesis in midges held at 25 °C: BTV virogenesis is evident in pools of *C. sonorensis* exposed to BTV-10 and BTV-2+10 at 7– 8 days after blood meal when held at 25 °C. BTV-2 remains near undetectable limits across all days. (**C**) BTV virogenesis in midges held at 20 °C: BTV virogenesis is evident in pools of *C. sonorensis* exposed to BTV-10 and BTV-2+10 at ~15 days after blood meal when held at 20 °C. BTV-2 remains near undetectable limits across all days. Please see the table within each figure for more details regarding regression analysis.

Then, we used these same virus stocks to expose *C. sonorensis* midges to an infective blood meal of either BTV-2, BTV-10 or BTV-2+10 at virus titers listed in Table 1. Midges were held at one of three temperatures (20, 25 or 30 °C) for the remainder of the experiment. Five *C. sonorensis* midges were collected in triplicate from each temperature and infection group every other day and were processed by BTV qRT-PCR to evaluate the presence of BTV nucleic acid. Midges exposed to BTV-10 or BTV-2+10 demonstrated signs of productive virogenesis (i.e., ΔCt values lower than day 0 infection levels) during the course of the experiment as early as day 4 post-infection in the higher temperature groups (Figure 2). In contrast, at no time point did the ΔCt values of midges exposed to BTV-2 drop below the baseline values, regardless of temperature (Figure 2).

At 30 °C, productive virogenesis was first detected on day 4 for BTV-10 and BTV-2+10 (Figure 2). For midges at 25 °C, productive BTV-10 virogenesis was first detected on day 10, and in the co-exposed group at day 8 (Figure 2). Midges held at 20 °C only demonstrated decreased BTV Ct values starting at days 15 and 16 for BTV-2+10 and BTV-10, respectively (Figure 2). The rate of virogenesis was determined from the slopes of linear regression analysis for each virus exposure group and temperature and data included in the analysis started from the upward trend of −ΔCt values. The slopes of BTV-10 at 30, 25, and 20 °C were 1.433, 0.2869, and 0.5987, respectively. Comparisons of slopes between temperature groups for BTV-10 demonstrated no statistical difference (*p* > 0.05, one-way ANOVA). The slopes of BTV-2+10 at 30, 25, and 20 °C were 1.694, 0.6120, and 0.4560, respectively. Comparisons of slopes for temperature groups for BTV-2+10 demonstrated a statistical difference between temperature groups at 30 and 20 °C (adjusted *p*-value < 0.0435, one-way ANOVA with Tukey’s post-hoc).

**Figure 3:**
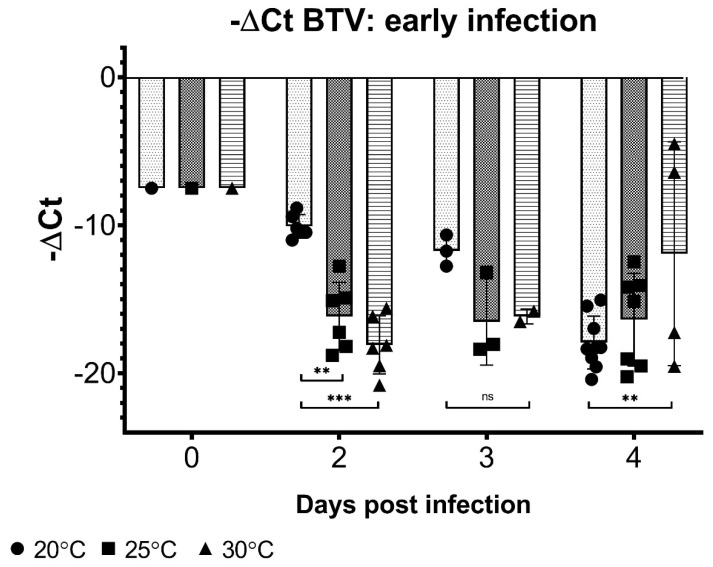
BTV −ΔCt values in midges following blood meal. Days 2–4 post-blood meal, *Culicoides* held at different temperatures demonstrate different rates viral RNA depletion, with cooler temperatures (20 °C) associated with slower decreases in BTV copy number compared to high temperatures. Day 0 –ΔCt values represent mean from pools collected immediately post-blood meal. Midges collected from all viruses (BTV-2, BTV-10, and BTV-2+10) are represented at each time point and undetectable BTV Ct values were not included in the analysis. Two-way ANOVA with Tukey’s post-hoc was used to analyze differences between temperatures, with *p* < 0.05 considered significant. ** Indicates *p* < 0.005, *** indicates *p* < 0.0001, and ns indicates *p* > 0.05/not significant.

Infection dynamics, as detected by viral RNA levels, are influenced by the temperature early after initial exposure. While BTV RNA levels rapidly reached near-undetectable limits in midges held at 30 °C by day 2 post-exposure, *Culicoides* held at 20 °C reached comparable BTV ΔCt values only at 4 days post-blood meal (Figure 3). Insects held at 25 °C also demonstrated slightly slower rates of BTV RNA disappearance than those at 30 °C, although they were largely comparable between the two upper temperatures. Normalized BTV Ct values for 20 °C midge pools were statistically lower (i.e., more virus still present) at day 2 post-blood meal than 25 or 30 °C midge pools (*p* < 0.003, two-way ANOVA with Tukey’s post-hoc), and significantly higher (i.e., less virus present) BTV ΔCt values at day 4 post-infection compared to 30 °C midges (*p* < 0.005).

**Figure 4:**
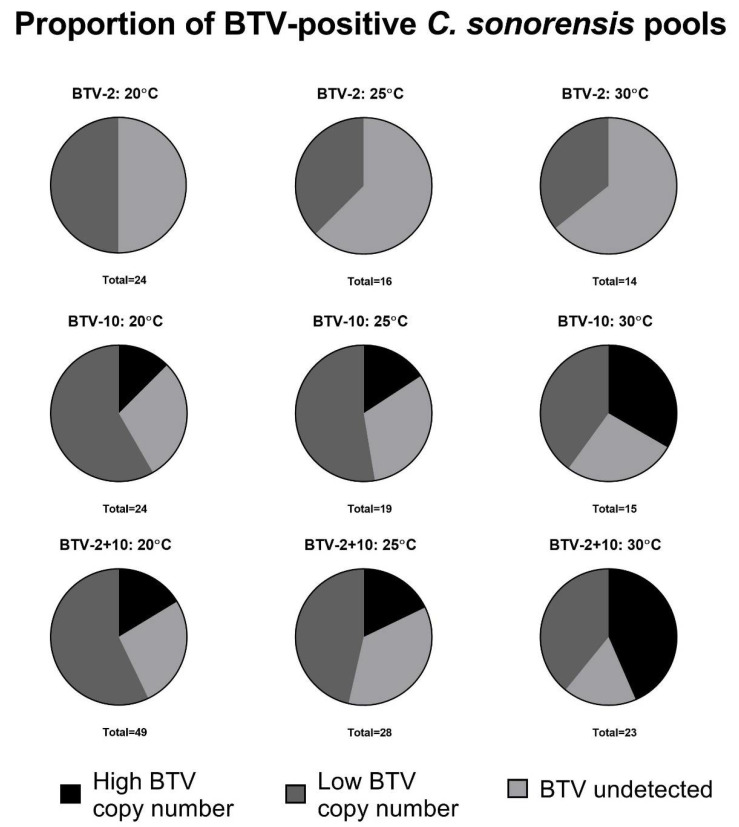
Proportion of BTV-positive *C. sonorensis* pools at each temperature. Pie charts represent the proportion of BTV-positive midge pools at each temperature across virus exposure conditions (BTV-2 only; BTV-10 only; BTV-2+10) when midges imbibed a blood meal containing a total MOI of ~1 × 10^5^ TCID50/mL. The total number of pools screened for BTV via qRT-PCR is noted beneath each pie chart. “High BTV copy number” indicates pools of midges where BTV −ΔCt values became greater than the day 0 –ΔCt value (−7.5), consistent with productive virogenesis. “Low BTV copy number” indicates pools of midges where BTV was detectable via qRT-PCR, but −ΔCt values were less than the day 0 –ΔCt value (−7.5). Productive virogenesis was not considered to have occurred in these pools. A greater proportion of midge pools demonstrate productive BTV virogenesis at 30 °C compared to lower temperatures. Insects infected with BTV-2 alone failed to demonstrate productive virogenesis regardless of incubation temperature.

Overall, a greater proportion of insect pools demonstrated productive virogenesis among 30 °C groups compared to 20 or 25 °C groups (Figure 4). Moreover, across our non-high titer experiments, BTV-2-exposed insects failed to generate increased BTV copy numbers following exposure, although BTV-2 was still detectable by qRT-PCR in many of our pools, particularly at early time points. BTV-2 Ct values became largely undetectable around the same time points that BTV-10 and BTV-2+10 began to reach levels indicative of productive virogenesis at each respective temperature.

Then, we attempted to infect smaller groups of *C. sonorensis* with higher titers of BTV-2 to see if midges were susceptible at higher doses of the virus. Interestingly, we found that some pools of midges did demonstrate productive virogenesis when exposed to titers 30- and 50-fold higher than our initial single-virus exposure experiments. At day 11, one pool from our highest dose (BTV-2 HI) had a BTV Ct value of 24.6, resulting in a ΔCt value of 2.98 (Table 2). consistent with successful BTV virogenesis. A similar finding was identified in a single pool from the BTV-2 LO group on day 14 (BTV Ct value of 22.9, resulting in ΔCt of 0.54). The virus was successfully isolated from this pool of midges as indicated by the generation of CPE on BHK-21 cells and subsequent confirmation via BTV qRT-PCR, confirming productive virogenesis in this pool of midges. Survival rates were similar between BTV-2 HI and BTV-2 LO groups maintained at 25 °C.

### 3.3. Temperature Effect on C. sonorensis Survival Rates

The longer a vector lives, the more likely it is to transmit infection. To establish whether BTV infection or temperature—or the interaction of these two variables—affected midge survival, we performed survival experiments in duplicate with each virus (BTV-2, BTV-10, BTV-2+10, negative) at each temperature.

**Figure 5:**
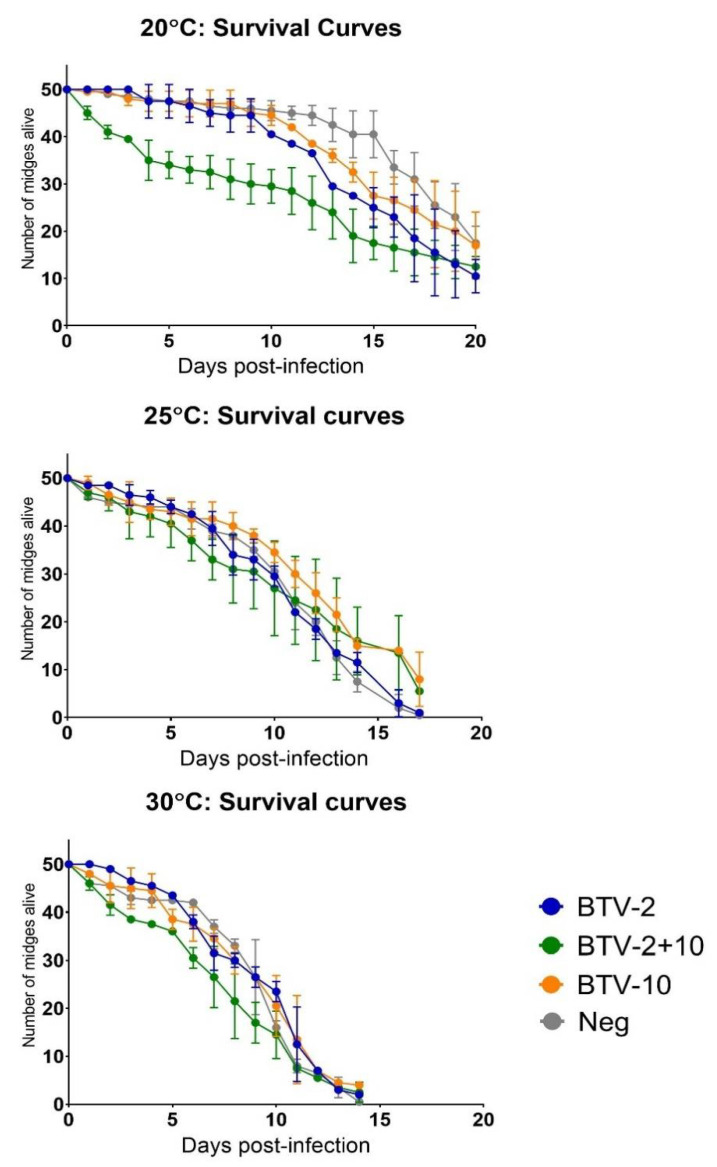
Survival of *C. sonorensis* at different temperatures. Survival groups (n = 50 per group) were infected in duplicate with the virus via blood meal (BTV-2, BTV-10, BTV-2+10 or negative) at the same titers as experimental groups. Survival groups were held at the respective temperatures (20, 25 or 30 °C) for the duration of the experiment and the number of surviving midges for each group was counted daily. Error bars depict the range at each time point.

Midges at 30 °C died earlier than those held at the lower temperatures, regardless of virus exposure status (Figure 5). Those held at 25 °C died a day or two later than those at 30 °C and midges held at 20 °C were still alive at 20 days post-infection. Survival rates of midges at 30 °C did not demonstrate significant differences between exposure status (*p* > 0.05, log-rank Mantel-Cox test). However, survival rates of midges at 25 and 20 °C did demonstrate significant differences between exposure status (*p*-value of 0.0004 and 0.0001 respectively, log-rank Mantel-Cox test). Most apparent in the experiment at 20 °C, while *Culicoides* with single-virus exposure survived at similar rates (*p* > 0.0083, log-rank Mantel-Cox test with Bonferroni adjustment), co-exposed midges died at a faster rate initially compared to un-exposed and singly exposed BTV-10 insects (*p*-value of 0.0001 and 0.0034 respectively, log-rank Mantel-Cox with Bonferroni adjustment). Insects that took a BTV-negative blood meal and were held at 20 °C survived the longest of all groups and had higher survival rates than BTV-exposed *Culicoides* held at the same temperature.

### 3.4. BTV-2 and 10 Plaque Genotypes

Pools of co-exposed midges from each temperature were prepared in triplicate for plaque assays every 4 days during the course of the experiment. Plaques were first isolated from *Culicoides* at 25 and 30 °C on day 7, and from *Culicoides* at 20 °C on day 11 (Table 3). Thereafter, plaques were detected from one or more pools of insects from all temperatures at each time point (until no midges were remaining at a respective temperature). Occasionally, infrequent plaques (e.g., <5 at lowest dilutions) were detected from pools of midges with low viral copy numbers, although these were not considered to be “productive virogenesis” due to the very low number of plaques.

**Figure 6:**
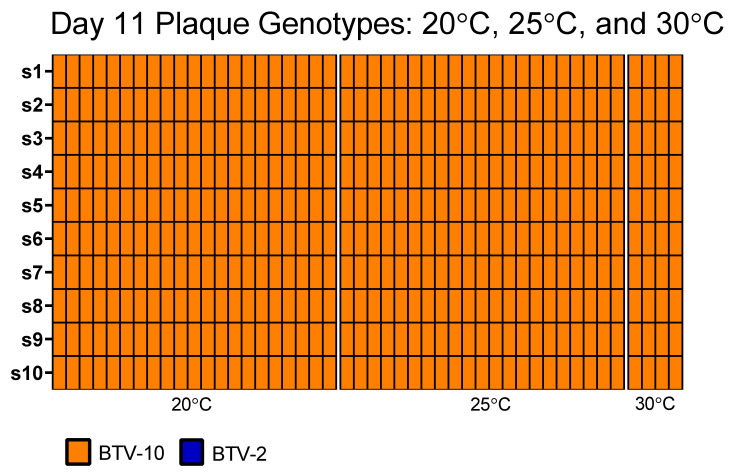
Genotype of plaques isolated from BTV-2+10 co-exposed midges at day 11 post-blood meal. Plaque genotypes from plaque isolated viruses from pools of BTV-2+10 exposed midges collected on day 11 post-infection as detected via amplicon-based sequencing. Each column represents the full 10 segments of an individual plaque (s1 through s10) in descending order. Only plaques where the complete genotype could be determined are depicted here. Plaques where sequencing reads were very low or where multiple segments did not receive reads are not included. In some cases where only segment 2 was not detected via amplicon sequencing, BTV serotype-specific qRT-PCR was used to determine the identity of the segment. Plaques isolated from midges held at different temperatures (20, 25, 30 °C) are demarcated by white margins.

Plaques were propagated once on BHK 21 cells and then selected plaques were prepared for genotyping via amplicon sequencing. As midges at all temperatures generated plaques by day 11, we randomly selected 23 plaques from 20 °C midges, 27 from 25 °C midges, and 9 plaques from 30 °C midges from day 11 for genotyping. Of the 59 total plaques screened from day 11, five samples were excluded from the analysis due to the low depth of coverage across all segments or low-level mis-mapping. Of the remaining plaques, six did not receive reads for both segments 2 and 3; two did not receive reads for segment 3 alone; and 35 plaques did not receive reads for segment 2 alone. Eleven plaques had sufficient coverage to identify the genotype of all 10 segments. The genotype of all detected segments was BTV-10 and no plaques contained any segments derived from BTV-2. For plaques where segment 2 alone was not successfully genotyped via the amplicon assay, serotype-specific BTV qRT-PCR was used to distinguish whether segment 2 was contributed by BTV-2 or BTV-10 [48]. All were genotyped as BTV-10 (Figure 6).

**Figure 7:**
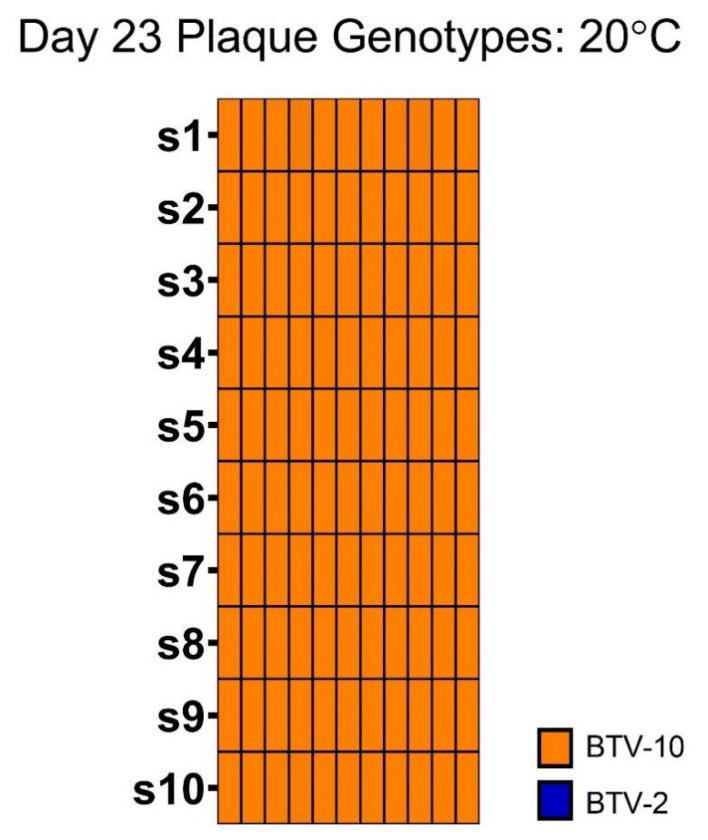
Genotype of plaques isolated from BTV-2+10 co-exposed midges at day 23 post-blood meal. Plaque genotypes from plaque-isolated viruses from pools of BTV-2+10 exposed midges collected on day 23 post-infection as detected via amplicon-based sequencing. Each column represents the full 10 segments of an individual plaque (s1 through s10) in descending order. Only plaques where the complete genotype could be determined are depicted here. Plaques where sequencing reads were very low or where multiple segments did not receive reads are not included. In some cases where only segment 2 was not detected via amplicon sequencing, BTV serotype-specific qRT-PCR was used to determine the identity of the segment.

As a follow-up and to determine whether reassortment might be more likely after a longer incubation period, we screened 12 randomly selected plaques from day 23 BTV-2+10 exposed midges that had been maintained at 20 °C. Of these, all plaques demonstrated at least 9/10 segments derived from BTV-10. One plaque was excluded from analysis due to low-level mis-mapping. A high percentage (50%) of plaques failed to generate reads aligning to either parent strain for segment 2. Subsequent screening via serotype-specific qRT-PCR demonstrated that segment 2 of all the plaques was derived from BTV-10 (Figure 7).

## 4. Discussion

Understanding how temperature affects virogenesis and reassortment among BTV strains is vital for our ability to accurately predict potential BTV incursions and epizootics, both in North America and worldwide. While temperature has a well-described effect on the extrinsic incubation period (EIP) of various vector-borne diseases including BTV, little is known regarding how or whether environmental factors such as temperature can affect the frequency of reassortment among segmented arboviruses [30,32,49,50,51]. This study, therefore, represents one of the first attempts to better characterize the impact of temperature on rates of reassortment in arthropod vectors.

Importantly, we did not detect reassortment between BTV-2 and BTV-10 in co-exposed midges, even though it has been previously demonstrated that these BTV strains can reassort in BHK 21 cells [37]. Therefore, the findings of this study allude to factors that may restrict reassortment between enzootic BTV strains, further highlighting the complexities of BTV evolution and ecology.

*Culicoides*-derived cells were susceptible to infection with BTV-2 and BTV-10 and both viruses demonstrated similar growth kinetics in this cell line. These findings provided an initial basis for us to suspect that *C. sonorensis* midges could support the replication of both BTV strains equally well. However, the *C. sonorensis* midges exposed in our study demonstrated much higher rates of infection with BTV-10 compared to BTV-2. We also suspect that the productive virogenesis detected in our midges exposed to BTV-2+10 predominantly reflects the replication of BTV-10, as implied by the findings of our plaque genotyping assay.

Two previous studies have assessed whether *C. sonorensis* midges are competent vectors for BTV-2, yet found differing results [52]. An initial study carried out shortly after BTV-2 was first detected in Florida in 1982 was performed using two strains of BTV-2, one of which was the BTV-2 OnaB 1982 strain [53]. This is putatively the same strain as that which was deposited at ATCC and subsequently used in our study. Consistent with our findings, very low rates of infection were found in *C. sonorensis* (~2% from the AA or Sonora colony in this early study) [53,54]. However, the viral titers used to infect midges in this experiment were not specified. Other studies have successfully infected *C. sonorensis* with a BTV-2 vaccine strain at a specified titer range of 6.0 and 5.5 log10 TCID50/mL [55].

Subsequently, Tanya et al. used *C. sonorensis* from an unspecified colony to investigate their competence for BTV-2 OnaB 1983 [52]. This isolate of BTV-2 was also detected in Ona, FL, but from a different year than the isolate used in the prior study. While the electropherotype of BTV-2 OnaB 1982 and BTV-2 OnaB 1983 were reportedly the same, no sequencing data currently exists to confirm this, and it is possible they are reassortant in non-segment 2 segments [56]. Tanya et al. found that BTV-2 OnaB 1983 was readily transmitted to sheep from infected midges, and that the oral infection rate of *C. sonorensis* was ~46% when blood meal BTV titers were 4.5 log10 [52]. This titer of BTV-2 is less than what was provided to midges in our experiments, and markedly less than the high titer doses we eventually used. Collectively, these conflicting findings highlight the numerous factors—both viral and otherwise—that can impact the likelihood of productive BTV infection following oral exposure to this virus.

Similar to early findings with BTV-2, our study highlights the role that vector species may play in the overall transmission and dispersion of various strains of BTV. It implicates diminished rates of BTV-2 infection in the *C. sonorensis*—the predominant BTV vector in North America—as a central reason for the failure of this particular serotype to become widely established in the US despite its long-term presence in certain regions [57]. Given that BTV-2 continues to circulate in the southeast US—where *C. sonorensis* is rare but other competent vectors such as *C. insignis* exist—it is likely that the vector species plays a key role in the circulation and range of BTV strains [13]. The expansion of *C. insignis* and reports of BTV-2 reassortment in BHK 21 cells and in the field allude to potential increases in the range of this serotype within North America [17,27,37,57]. A better understanding of the likelihood of BTV reassortment in *C. insignis*—and other potential BTV vector species—is fundamental as we approach questions involving BTV evolution and ecology in North America.

Viruses must overcome a variety of barriers to successfully infect an arthropod host and eventually become transmissible. These include the mesenteron infection barrier, the mesenteron escape barrier, the salivary gland infection barrier, and the salivary gland escape barrier, although the salivary infection barrier is reported to not exist in *Culicoides* [58,59,60]. *Culicoides* are also believed to have a dissemination barrier that restricts BTV replication beyond the gut cells [59]. Our findings indicate that the BTV-2 strain used here may only rarely be able to overcome the mesenteron escape barrier in *C. sonorensis* from the AK colony, given that BTV ΔCt values never indicated productive BTV-2 virogenesis at biologically relevant oral infection titers. Interestingly, the VP7 protein (encoded by segment 7)—which is responsible for viral core particle binding to *Culicoides* cells—has an identical amino acid sequence between the BTV-10 and BTV-2 strains used in our study [61,62]. This implies that additional factors beyond successful VP7-mediated cell binding likely modulate the BTV-2 ability to effectively infect *C. sonorensis*. Additional studies with intrathoracic inoculation of midges are warranted to better understand these barriers to infection.

It remains unclear why midges were successfully infected with BTV-2 only at high viral titers, and further studies are indicated to better understand this finding. It is possible that a BTV-2 variant only present at very low levels in the viral milieu was more successful at infecting *C. sonorensis*. Therefore, only when midges were exposed to much higher titers of the virus was this variant present and able to infect the midges. Alternatively, higher titers of BTV-2 may have been more likely to overwhelm intrinsic RNAi or other immune mechanisms that might prevent successful midge infection at lower doses of this particular BTV strain.

We observed differing rates of *Culicoides* death at different temperatures, which was a uniform finding across all viral exposure status groups. This is consistent with findings from other vector studies, predominantly in mosquitoes, where higher incubation temperatures drive more rapid vector mortality [51,63]. Interestingly, at our lowest temperature (20 °C), we detected distinctions in mortality rate between non-exposed, singly-exposed, and co-exposed midges. Similar findings have been noted subsequent to arboviral infections in mosquito vectors. These trends were only noted at our lowest incubation temperature, which is noteworthy [64,65,66]. Increased mortality at 20 °C may be associated with reduced ability to control viral infection at lower temperatures. Notably, this trend was present among midges exposed to BTV-2 as well as BTV-10, particularly from day 10 forward. The most dramatic mortality rate was detected in midges co-exposed with BTV-2+10. Even though BTV-2 singly exposed midges did not have pools indicative of productive virogenesis when exposed to lower BTV-2 titers, decreased survival rates were nonetheless observed in these groups, suggesting that this could be due to the exposure to a virus or the growth medium used for BTV amplification (Figure 5). It is important to recognize that single and co-exposure groups had equivalent amounts of growth medium during blood feeding. Therefore, we suspect that viral exposure was the more prominent factor affecting survival.

Collectively, these findings suggest that there may be an important interaction between exposure status and temperature. Despite the rare incidence of successful BTV-2 infection in midges, it appears that exposure to BTV-2 at sub-infective doses may nonetheless affect midge survival at cooler temperatures, alluding to other potential underlying causes for absence of robust BTV-2 virogenesis at biologically relevant infection titers. This finding has implications for understanding the complex interactions of vector competence, environmental temperature, and overall survival rates, and will contribute to our ability to better model and predict BTV outbreaks and incursions in the face of climate change.

Finally, we noted that a greater proportion of midges held at 30 °C demonstrated high BTV copy numbers, indicating increased virogenesis in these insects. Although we did not directly measure how high rates of BTV virogenesis affected vector competence or infectious titer, it stands to reason that greater virogenesis may be associated with increased BTV transmission and—in the case of BTV strains that can successfully replicate in the vector—reassortment. Collectively, these findings highlight the complexities of virus-vector interactions that underlie bluetongue ecology in North America.

## Figures and Tables

**Table 1:** Groups of *C. sonorensis* exposed to BTV in Experiments 1 and 2.

	BTV-2			BTV-10			BTV-2+10			Negative			
Temperature	20 °C	25 °C	30 °C	20 °C	25 °C	30 °C	20 °C	25 °C	30 °C	20 °C	25 °C		30 °C
Experiment ID	2	2	2	2	2	2	1	1	1	-	1	2	-
Number of midges per container	n = 150	n = 165	n = 136	n = 150	n = 150	n = 150	n = 454	n = 614	n = 724	-	n = 312	n = 252	-
	n = 150	n = 153	n = 144	n = 150	n = 150	n = 185	n = 534	n = 741	n = 556				
Mean Bloodmeal BTV Titer	1.02 × 10^5^	1.02 × 10^5^	1.02 × 10^5^	1.06 × 10^5^	1.06 × 10^5^	1.06 × 10^5^	BTV-2: 5.1 × 10^4^	BTV-2: 5.1 × 10^4^	BTV-2: 5.1 × 10^4^	-	-		-
(TCID50/mL)							BTV-10: 5.3 × 10^4^	BTV-10: 5.3 × 10^4^	BTV-10: 5.3 × 10^4^				
Survival groups (Exp. 2 only)	n = 50 in duplicate	n = 50 in duplicate	n = 50 in duplicate	n = 50 in duplicate	n = 50 in duplicate	n = 50 in duplicate	n = 50 in duplicate	n = 50 in duplicate	n = 50 in duplicate	n = 50 in duplicate	n = 50 in duplicate		n = 50 in duplicate

**Table 2:** ΔCt values for pools of midges following exposure to higher titers of BTV-2. ΔCt is calculated as the difference between mean BTV Ct values and *cox*1 Ct values for each sample. Day 0 values represent the ΔCt value in midges (n = 5) collected immediately following ingestion of BTV-2 spiked blood meal. Pools of n = 5 midges were subsequently collected and screened on days 7, 11, and 14. “UND” indicates that the BTV Ct value was beyond the limit of detection in a pool of midges. Virus isolation was performed on one pool of midges collected at day 14 (indicated by *) for both the BTV-2 LO and HI groups. ^˄^ Indicates that the virus was successfully isolated from midges (day 14, BTV-2 LO). For these experiments, BTV virogenesis was considered to occur when *cox*1-normalized BTV Ct values approached the baseline day 0 BTV ΔCt level.

	BTV-2 LO (30-Fold Greater): ΔCt				BTV-2 HI (50-Fold Greater): ΔCt			
Day 0	−1.78				−2.10			
Day 7	13.84	19.27	19.14		9.66	17.17	15.64	
Day 11	11.98	13.58	16.53		UND	UND	2.98	
Day 14	16.19	14.36	14.84	0.54 *^,˄^	19.22	UND	17.38	UND *

**Table 3:** BTV plaque isolations. Pools of midges (n = 10 per pool) were homogenized in triplicate (A, B, C) and used to inoculate BHK 21 cells for plaque isolation at days 3, 7, 11, 15, 19, and 23 post-blood meal. Dashes (−) indicate replicates where no plaques were identified. Pools that produced ≥5 plaques are denoted by (+), and those that produced plaques rarely (<5 plaques at lowest dilution) are indicated by (+/−). Days where no surviving insects were available to perform plaque assays are (n/a).

	20 °C			25 °C			30 °C		
Days Post-Infection	A	B	C	A	B	C	A	B	C
3	−	−	−	−	−	−	−	−	−
7	−	−	−	+/−	−	+	−	−	+
11	+	+/−	+/−	+	+/−	+/−	+	+	+/−
15	+	+	+/−	+	+/−	+/−	n/a	n/a	n/a
19	−	−	+	n/a	n/a	n/a	n/a	n/a	n/a
23	+	−	−	n/a	n/a	n/a	n/a	n/a	n/a

## Data Availability

The data presented in this study are available on request from the corresponding author.

## Supplementary Materials

The following are available online at https://www.mdpi.com/article/10.3390/v13061016/s1, Figure S1: Comparative efficiencies of qPCRs: BTV vs cox1, Table S1: Amplicon assay primers.
