# Supplementary material for: Exposure of Culicoides sonorensis to Enzootic Strains of Bluetongue Virus Demonstrates Temperature- and Virus-Specific Effects on Virogenesis
Source: Viruses. 2021 May 28;13(6):1016. doi: 10.3390/v13061016 (PMC8228769; doi:10.3390/v13061016)
Supplement: Supplementary file 1 [file viruses-13-01016-s001.zip › viruses-1149742-supplementary.pdf]

Supplementary Materials

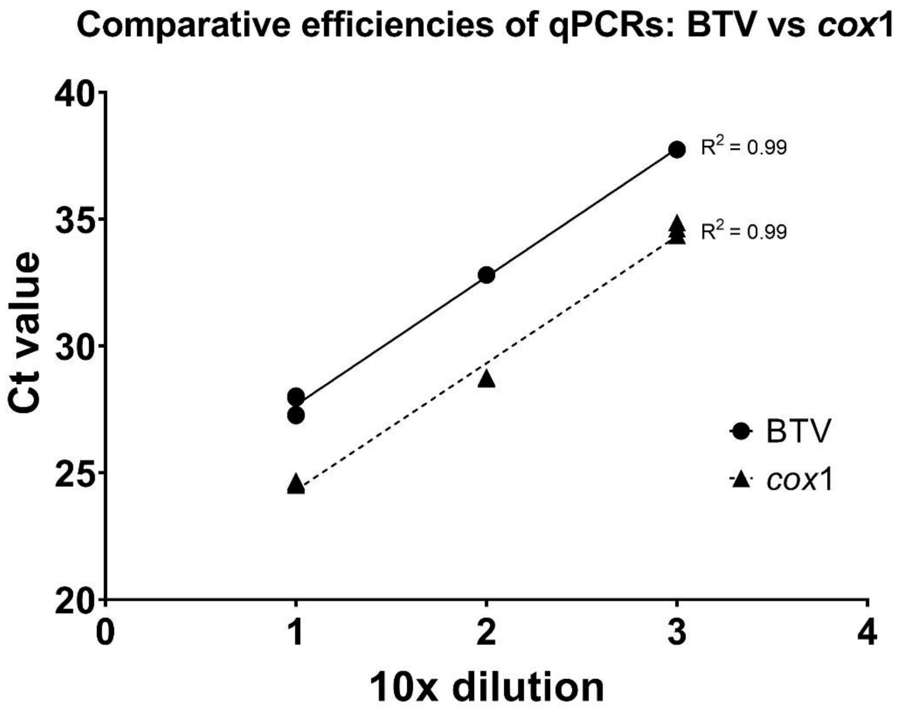

Figure S1. Comparative efficiencies of BTV (segment 10) and *Culicoides* mitochondrial cytochrome oxidase 1 gene (*cox*1) RT-PCR after DNase treatment. Ten-fold dilutions were prepared of a pool of BTV-infected midges, and each dilution was screened in triplicate for each target. Linear regressions and R^2 values were prepared using Graph Pad Prism 8.0.

Table S1. Amplicon Assay Primers. Round-one PCR of amplicon assay, BTV-specific primer sequences and concentrations. Final primer cocktail concentration (in μM) is shown for each primer. D11 represents the concentration used for plaques screened from midges collected on day 11 post-blood meal; D23 represents the concentrations used for plaques screened from midges collected on day 23 post-blood meal.

	Sequence	D11, μM	D23, μM
**S1_560F**	TCC AGG GGA ATA GAG ATT TAT C	0.13	0.12
**S1_1033R**	TCG TGC GAG CCY AAW TTT TG	0.13	0.12
**S2_1210F**	TGG CGA TGT KTA CTT YAC MTT GCG	0.10	0.11
**S2_1601R**	GCA TCY TTY TCG AAA TCG ATT GTA AG	0.10	0.11
**S3_2282F**	TMC AGT TYC GAG CGG CTT TAA G	0.08	0.10
**S3_2684R**	GAG CGA TTG GGT GAT GTC CA	0.08	0.10
**S4_1484F**	TCG TGG GCG ATG AAT TTT GCT	0.08	0.08
**S4_1961R**	TCA CCT AGC AGT CAC GCA TTA TAA G	0.08	0.08
**S5_177F**	TCG ATG ATY GCA GCA ACT GAT G	0.13	0.08
**S5_587R**	TGT GCT GTC CAC GAA TGC CAA	0.13	0.08
**S6_715F**	TAG GCG GCR TCW GAA GAA GTG	0.13	0.10
**S6_1099R**	YGG GAT CTT AAA YYT CAT CAT YAC	0.13	0.10
**S7_246F**	TTT TGG ACC GAT ATC GCC AGA	0.08	0.05
**S7_701R**	TGT CCA TCC CAC GCT ATA ATG C	0.08	0.05
**S8_594F**	TTG GAT GAW GAG GCC AAA GAG AT	0.08	0.09
**S8_1048R**	CTT AGA GAC AAA AGC AAC ACG CT	0.08	0.09
**S9_455F**	TAC GGT ACG AAG ATT GAT GTT TAC AG	0.10	0.09
**S9_902R**	TTC CAA TGC GGA TCT CCA GTT G	0.10	0.09
**S10_184F**	TAA ATY CTG GAC AAA GCG ATG TC	0.08	0.07

**S10_549R**	ACT YTT TGC GCA AAC CAT CAT CA	0.08	0.07
